# Silicon in action: Between iron scarcity and excess copper

**DOI:** 10.3389/fpls.2023.1039053

**Published:** 2023-02-03

**Authors:** Dragana Nikolić, Dragana Bosnić, Jelena Samardžić

**Affiliations:** Laboratory for Plant Molecular Biology, Institute of Molecular Genetics and Genetic Engineering (IMGGE), University of Belgrade, Belgrade, Serbia

**Keywords:** iron deficiency, copper toxicity, cross-talk between elements, stress alleviation, silicon

## Abstract

Essential micronutrients belonging to the transition metals, such as Fe and Cu, are indispensable for plant growth and stress tolerance; however, when present in excess, they can become potentially dangerous producers of reactive oxygen species. Therefore, their homeostases must be strictly regulated. Both microelement deficiencies and elevated concentrations of heavy metals in the soil are global problems that reduce the nutritional value of crops and seriously affect human health. Silicon, a beneficial element known for its protective properties, has been reported to alleviate the symptoms of Cu toxicity and Fe deficiency stress in plants; however, we are still far from a comprehensive understanding of the underlying molecular mechanisms. Although Si-mediated mitigation of these stresses has been clearly demonstrated for some species, the effects of Si vary depending on plant species, growing conditions and experimental design. In this review, the proposed mechanistic models explaining the effect of Si are summarized and discussed. Iron and copper compete for the common metal transporters and share the same transport routes, hence, inadequate concentration of one element leads to disturbances of another. Silicon is reported to beneficially influence not only the distribution of the element supplied below or above the optimal concentration, but also the distribution of other microelements, as well as their molar ratios. The influence of Si on Cu immobilization and retention in the root, as well as Si-induced Fe remobilization from the source to the sink organs are of vital importance. The changes in cellular Cu and Fe localization are considered to play a crucial role in restoring homeostasis of these microelements. Silicon has been shown to stimulate the accumulation of metal chelators involved in both the mobilization of deficient elements and scavenging excess heavy metals. Research into the mechanisms of the ameliorative effects of Si is valuable for reducing mineral stress in plants and improving the nutritional value of crops. This review aims to provide a thorough and critical overview of the current state of knowledge in this field and to discuss discrepancies in the observed effects of Si and different views on its mode of action.

## 1 Introduction

Silicon (Si) is widely accepted as a beneficial element in plants. Over the past twenty years, numerous investigations have been carried out to elucidate the mechanisms of action of Si in plants exposed to various biotic and abiotic stress conditions. It has been acknowledged that Si can improve plant nutrition, and further research is being done to clarify how Si affects plants that are exposed to nutritional deficiencies or metal toxicity. (Ali et al., 2020; Khan et al., 2021).

Under the conditions of metal toxicity, the co-deposition of Si with metals in the root cell wall helps to reduce the uptake and translocation of metal to the aerial parts of plants. In shoots, Si mitigates the harmful effects of excess metals by sequestering them into the leaf vacuoles and reducing oxidative stress by enhancing antioxidative defense (Ali et al., 2020). On the other hand, under nutritional deficiency, the role of Si in the formation and use of the apoplastic metal pools is recognized (Pavlovic et al., 2013; Pavlovic et al., 2021). Silicon enhances uptake of the deficient nutrient in root and increases its root-to shoot translocation.

In this article, the findings on the influence of Si on plants encountering inadequate availability of Fe and Cu are reviewed and discussed. These essential microelements are crucial for many cellular processes in plants, such as photosynthesis, respiration and antioxidant defense. On the other hand, these transition metals are highly reactive and, when present in excess, lead to the accumulation of reactive oxygen species (ROS) and oxidative damage to cellular components (Halliwell, 2006). Thus, their homeostases must be strictly regulated. Both microelement deficiencies and high concentrations of heavy metals in soil are widespread problems that decrease the nutritional value of crops and negatively affect human health (McLean et al., 2009). Silicon fertilization is considered a promising measure to improve crop resilience, and research into the ameliorative potential of Si is therefore increasingly appreciated. The effects of Si on Cu homeostasis in plants have been studied primarily in the context of Cu toxicity, while its role in relation to Fe has been studied mainly in the context of Fe deficiency. Silicon has been reported to alleviate symptoms of both stresses in different plant species. However, the effects of Si vary depending on the plant species, growing conditions and experimental design. Although considerable progress has been made in the last decade, we are still far from fully understanding the molecular mechanisms underlying the effects of Si. This review aims to provide a thorough and critical overview of the current state of knowledge in this field and to discuss discrepancies in the observed effects of Si and different views on its mode of action.

### 1.1 The role of iron and copper in plants

Microelements such as iron (Fe), copper (Cu), manganese (Mn) and zinc (Zn) are essential for plants, and their functions are connected to crucial metabolic processes, growth and yield, stress tolerance and disease resistance. Both supra-optimal and suboptimal concentrations of micronutrients in soil negatively affect plants. Iron and copper are transition metals that are redox active and can be found in two oxidation states (Fe^2+^ and Fe^3+/^Cu^+^ and Cu^2+^). The ability to change their oxidation states allows them to participate in many important biochemical processes in cells such as photosynthesis and respiration. Conversely, their readiness to fluctuate between reduced and oxidized forms makes them potentially dangerous producers of reactive oxygen species (ROS) *via* the Fenton reaction, and inducers of oxidative stress (Halliwell and Gutteridge, 1984; Halliwell, 2006).

Despite its usually high abundance in the soil, Fe is the third most limiting nutrient in plants as a result of its low availability, especially in alkaline and calcareous soils (Rout and Sahoo, 2015). Iron is indispensable in many vital cellular processes in plants such as photosynthesis, respiration, DNA synthesis, nitrogen reduction, antioxidative defense, hormone biosynthesis. If an adequate amount of Fe is not available to plants, Fe deficiency (Fe interveinal chlorosis) occurs, particularly in the youngest leaves. Since the main source of Fe for humans are plants, its poor bioavailability and low content in plants are often related to common nutritional disorders in humans, including anemia, which affects nearly 25% of the world’s population (McLean et al., 2009).

Cu readily oscillates between its oxidized Cu^2 +^ and reduced Cu^+^ state, making it an ideal protein cofactor in redox reactions. Copper is a cofactor in Cu proteins that play a significant role in cell metabolism, with over a hundred such proteins found in plants (Krämer and Clemens, 2006). Photosynthesis and respiration are the two most important processes in plants based on electron transfer reactions involving Cu-proteins (Marschner, 1995). The most important Cu-proteins are plastocyanin, cytochrome-c-oxidase, Cu/Zn superoxide dismutase (SOD), laccases, etc. (Yruela, 2009).

### 1.2 Uptake and distribution of Fe

To obtain Fe from the rhizosphere, higher plants have developed two major strategies, designated as Strategy I and II (Römheld and Marschner, 1986). Strategy I, or a reduction-based strategy, is present in most dicotyledonous plants and is well-studied. It involves three participants. The first is a proton pump, the plasma membrane H^+^ATPase (P-ATPase), which increases the solubility of Fe^3+^ in the rhizosphere. Soluble Fe^3+^ then enters the apoplast where it is reduced to Fe^2+^ by plasma membrane ferric chelate reductase FRO2 (ferric reduction oxidase 2). After reduction, Fe^2+^ is transferred by the high-affinity iron-regulated transporter1 (IRT1) into root epidermal cells (Marschner, 1995; Vert et al., 2002; Santi and Schmidt, 2009). Strategy II is restricted to the family Poaceae and is also known as a chelation-based strategy (Marschner, 1995). It is based on the biosynthesis of chelating compounds, phytosiderophores (PS), a class of mugineic acid compounds, and their secretion in the rhizosphere. PS chelate Fe^3+^ and PS-Fe (III) complexes are transported into root cells by yellow stripe and the yellow stripe-like (YS/YSL) family of transporters (Morrissey and Guerinot, 2009).

Certain graminaceous species (rice) use a combination of strategies I and II to acquire Fe. Following symplastic entry, Fe is loaded in the xylem sap and translocated into the shoots through the transpiration stream. Organic acids, especially citrate, are the main metal-chelating compounds in the xylem. Source-to-sink transport of Fe *via* phloem has been less studied (Curie and Briat, 2003). One of the most important molecules possessing a Fe-chelating affinity that has been identified in phloem sap is nicotianamine (NA) (Carrillo and Borthakur, 2021; Hanikenne et al., 2021). Nicotianamine is a non-proteinogenic amino acid synthesized by nicotianamine synthase (NAS) that is capable of binding transition metals with high affinity. Nicotianamine’s critical role in metal homeostasis in plants has mostly been elucidated for Fe and Cu (Curie et al., 2009). The NA-metal complex enters cells *via* a YSL transporter. The main role of NA and YSL is lateral transport and metal distribution in leaves, as well as the remobilization of metals during senescence and their storage in seeds (Takahashi et al., 2003; Curie et al., 2009). In graminaceous plants, in addition to NA, phytosiderophores play an important role in internal metal transport (Nishiyama et al., 2012; Carrillo and Borthakur, 2021).

### 1.3 Uptake and distribution of Cu

Variations in the copper content in plants are influenced by the bioavailability of Cu in the soil, but also by the presence of other essential elements. The bioavailability of Cu depends on the physical and chemical soil properties, primarily the pH, as well as on biological processes in the rhizosphere. Copper is found mainly immobilized in minerals, and a smaller percentage of total Cu is present in soil solution in the form of a divalent cation (Kabata-Pendias and Pendias, 2001). The mechanism of Cu uptake in dicots is similar to Fe uptake and entails ion reduction on the plasma membrane and adoption of Cu^+^, its reduced form (Ryan et al., 2013). Members of the FRO family catalyze the reduction of Fe^3+^ and Cu^2+^; FRO2 in the root is most important for Fe homeostasis while FRO4 and FRO5 are involved in Cu homeostasis maintenance (Robinson et al., 1999; Bernal et al., 2012). Transport of the reduced, Cu^+^ form is mainly mediated by high-affinity transporters belonging to the conserved copper protein transporter (COPT) family, which is present in all eukaryotes (Puig et al., 2002). The most important transporter for root uptake of Cu^+^ is plasma-membrane COPT1 (Sancenón et al., 2004).

After being taken up by root cells, Cu ions are loaded into the xylem by P-type ATPases or HMA (heavy metal ATPases) (Shikanai et al., 2003; Abdel-Ghany et al., 2005). In the xylem, most copper is bound to NA, which allows translocation of Cu. Nicotianamine is also important for Cu distribution within the shoot *via* the phloem pathway (Pich and Scholz, 1996; Takahashi et al., 2003). In cells, the high reactivity of reduced copper requires the involvement of Cu chaperones to prevent the formation of ROS and unintended interactions with other cellular components (Harrison et al., 1999).

### 1.4 Iron deficiency and copper toxicity as important nutrient disorders in plants

#### 1.4.1 Iron deficiency in plants

The first visible symptom of Fe deficiency in plants is chlorosis, which is characterized by impaired photosynthesis. The high demand for Fe in photosynthesis has led to the development of mechanisms to reduce the stress caused by Fe deficiency. Under Fe-limiting conditions, all plants respond by 1) increasing Fe uptake; 2) modifying metabolism; 3) increasing the efficiency of Fe utilization; 4) remobilizing stored Fe. In plants employing strategy I, many morphological and biochemical adaptations have evolved to increase the capacity for Fe uptake. Morphological adaptations include swelling of root tips and the formation of lateral roots, root hairs and transfer cells that increase root surface area. Biochemical changes include up-regulation of a plasma membrane FRO2 and an IRT1, increased proton extrusion and exudation of organic compounds such as carboxylates, flavins and phenolic compounds (Abadía et al., 2011). The advent of integrated omics has enabled a comprehensive analysis of plant responses to low Fe at the molecular level. Conversely, several studies mostly based on forward and reverse genetic approaches were carried out, putting into the foreground transcription factors as master controllers of Fe homeostasis (Gao et al., 2019).

Enhanced production of carboxylic acids (citrate and malate) and NA is observed in Fe-deficient plants. These low molecular weight compounds are involved in Fe mobilization and distribution to the most Fe-demanding plant structures, chloroplasts and mitochondria (Abadía et al., 2002).

#### 1.4.2 Copper toxicity in plants

The toxic effects of Cu on plants were recognized before its essentiality was proven. Phytotoxicity is observed at the morphological, physiological and molecular levels at a concentration of Cu of 20 µg/g dry weight and greater (Marschner, 1995). Copper primarily accumulates in the root, so its changes and harmful effects are more pronounced in this plant organ and manifest as a progressive reduction in growth, development, branching and formation of dark coloration of the root. In the aboveground part, the symptoms of toxicity include growth inhibition, chlorosis and leaf necrosis, and changes in reproductive organs that lead to a reduction in fruit size and seed germination rate (Yruela, 2009). Excess Cu has an antagonistic effect on the uptake and translocation of other essential elements such as Fe, Zn and Mn (Ouzounidou, 1994; Schmidt et al., 1997; Michaud et al., 2008). At the cellular level, Cu has multiple negative effects on vital processes, such as photosynthesis and respiration (Marschner, 1995; Yruela, 2009). It also causes changes in the permeability of the cell membrane and leakage of cellular contents (De Vos et al., 1989; Murphy et al., 1999; Demidchik et al., 2014). High-affinity interactions between Cu and proteins occur directly or by displacing other essential cofactors from their sites, thus inhibiting the catalytic, structural or transport functions of the proteins (Van Assche and Clijsters, 1990).

Excess redox-active Cu ions induce the production of ROS and oxidative stress in cells. ROS have many effects on macromolecules, causing nucleic acid damage, enzyme inhibition, protein oxidation and lipid peroxidation, leading to the activation of programmed cell death (Sharma et al., 2012).

Plants have developed different mechanisms for toxic metal tolerance that are oriented toward reducing the accumulation of reactive ions to suppress direct damage to cellular structures. External mechanisms are aimed at decreasing metal uptake by forming ectomycorrhiza or by exudation of Cu-complexing chelators. Metal retention in the root is a mechanism that limits translocation to the aboveground part, thus preventing a negative impact on photosynthetic organs (Hall, 2002; Krämer and Clemens, 2006). Internal, cellular mechanisms of defense against Cu toxicity include immobilization of the metal in the cell wall, limited influx through the cell membrane and enhanced efflux from the cell. Mechanisms of metal detoxification include the formation of metal-ligand complexes and their deposition in vacuoles or trichomes. Metal binding to organic acids and sequestration in vacuoles is an important tolerance mechanism, which is particularly evident in metal-hyperaccumulating plants. In addition, the accumulation of free amino acids is an active response of plants to heavy metal stress. Amino acids such as histidine (His) and NA are potent Cu chelators that play a crucial role in protection against the toxicity of this metal (Hall, 2002; Mijovilovich et al., 2009; Anjum et al., 2015). Compounds containing thiol groups such as metallothioneins and phytochelatins have a strong affinity for Cu and therefore play an important role in detoxification (Hall, 2002).

#### 1.4.3 Interdependence of Fe and Cu homeostases in plants

Dual nature of essential but highly reactive micronutrients belonging to the transition metals, such as Fe, Cu, Mn and Zn requires tight regulation of their homeostasis (Rai et al., 2021). Moreover, their homeostases are interdependent and linked by a complex interplay (Hanikenne et al., 2021). These microelements share and compete for the same ion transporters, ligands, metal-binding proteins and storage pools. Therefore, the deficiency or excess of one micronutrient can alter the uptake and status of the others. Even though the molecular mechanisms of maintaining metal homeostasis for individual metals are being increasingly understood, the presumed cross-interactions along these pathways, which might take place at various points, remain mostly unknown. Plants may alternatively use Fe- or Cu-containing proteins to catalyze the same biochemical reactions, depending on the bioavailability of each of these metals. For example, Fe-containing superoxide dismutases (FeSODs) can perform the same functions as performed by Cu-containing SODs (CuSODs). The replacement of *Arabidopsis* Cu/ZnSOD with the FeSOD counterpart, under conditions of Cu scarcity, likely occurs to save Cu for essential cuproproteins such as plastocyanin. Mineral deficiency or excessive metal uptake can disturb regular plant physiology and metabolism, causing oxidative stress and requiring alterations in mineral uptake (Rai et al., 2021). Uptake of both Fe and Cu is mainly regulated at the transcriptional level. Some of the metal transporters can provide both Fe and Cu entries. The COPT family of transporters is predominantly responsible for the transport of Cu, but during the severe deficiency of Cu these transporters can also transport Fe (Kastoori Ramamurthy et al., 2018). Members of the Yellow Stripe-Like (YSL) family have been implicated in the short- and long-distance transport of multiple micronutrients complexed with NA (Carrillo and Borthakur, 2021). It has been shown that YSL2 transports both Fe and Cu and its expression is regulated by the levels of these metals in the growth medium (DiDonato et al., 2004).

## 2 Influence of Si on plant Fe status and Fe deficiency response

Despite the considerable published data on the beneficial effects of Si on the reinforcement of plant defenses against different biotic and abiotic stresses, there is some disagreement regarding the influence of Si on Fe status in plants and the alleviation of Fe deficiency. While Si-dependent mitigation of Fe deficiency stress has been demonstrated in some species, including cucumber (*Cucumis sativus*), soybean (*Glycine max*) and barley (*Hordeum vulgare, Hordeum marinum*) (Gonzalo et al., 2013; Pavlovic et al., 2013; Nikolic et al., 2019; Ksiaa et al., 2022), in several other studies, the absence of positive effect or the opposite effect was described (Bityutskii et al., 2010; Gonzalo et al., 2013; Hernández-Apaolaza et al., 2020). Such inconsistencies have even been reported in the same species.

Silicon-dependent amelioration of Fe deficiency was demonstrated first in cucumber and pumpkin (*Cucurbita pepo*) (Strategy I plants) by Bityutskii et al. (2010). The same authors reported that Si failed to improve tolerance to Fe shortage in Strategy II plants such as maize (*Zea mays*) and barley. Conversely, Nikolic et al. (2019) showed that Si alleviated Fe deficiency in barley by diminishing chlorosis and biomass loss of the youngest leaves, the plant organs that are most severely affected by this type of stress. *Sorghum bicolor* is another graminaceous plant in which Si supply was beneficial during Fe deficiency stress (Teixeira et al., 2020). In contrast, it has been reported that Si enhances Fe deficiency symptoms in rice (Becker et al., 2020). A notable decrease in the chlorophyll content was observed in Fe-deprived rice after the addition of Si, but without statistically significant changes in Fe concentration in shoots (Becker et al., 2020).

An inconsistent effect of Si in Fe-deficient plants was demonstrated even in the same species by different research teams. Alleviation of this nutritional disorder by Si supply was clearly shown in cucumber, the Chinese long variety (Pavlovic et al., 2013). Silicon addition mitigated chlorosis and dry weight loss in cucumber roots and shoots. On the other hand, in the study of Gonzalo et al. (2013), although Si delayed some Fe-deficiency symptoms in cucumber (cv. Ashley), such as a decrease in stem dry weight, stem length and node number, it did not alleviate leaf chlorosis. In another study it was noted that the exacerbation of Fe-deficiency symptoms in the same cucumber cultivar after the addition of Si. Silicon treatment of stressed plants led to a reduced leaf Fe concentration accompanied by increased ROS and lipid peroxidation levels in young leaves (Hernández-Apaolaza et al., 2020). Nonetheless, the negative effect of Si did not affect chlorophyll concentration and the dry weight of young leaves.

The disparities concerning the effect of Si on plant Fe status and tolerance of Fe deficiency stress could be ascribed to variations in growth conditions and differences in the experimental design. Aside from diverse plant species or cultivars, different authors applied dissimilar Si and Fe concentrations, Fe chelators, and growth media with different compositions and pH. The influence of the pH of the nutrient solution on the Si effect on plants deprived of Fe has been estimated in cucumber and rice (Bityutskii et al., 2018; Martín-Esquinas and Hernández-Apaolaza, 2021). Also, Gonzalo et al. (2013) have shown the importance of the type of Fe chelating agent and Si concentration. When Fe is added as a low stability Fe(III)-EDTA complex, in an alkaline calcareous solution, Fe-Si precipitates may form, especially at high Si concentrations. Under these conditions, Fe retention over the amorphous silica may reduce the availability of Fe to plants. The Si-Fe co-precipitation is avoided when a stronger chelating agent, Fe(III)-HBED, is used.

Also, important factors that could impact Si action are the plant growth stage, Fe-sufficient pretreatment, the duration and the severity of Fe deficiency, as well as the timing of Si addition. It is also unknown how growth conditions, such as the light regime and intensity, humidity, etc., can affect the influence of Si on Fe uptake and translocation.

Considering the above, for successful application in agriculture, it is advisable to evaluate new Si fertilizers for use on the specific crops and cultivars and growth conditions. Most of the published research was performed on plants grown in a nutrient solution. These results could be translated to plant production in hydroponics, which is becoming widely accepted. However, more attention should be paid to the effects of silicon on the uptake of heavy metals in soil-grown plants. More pot and field trials on the application of silicon in different soil types are needed. The alteration of soil properties such as pH, redox potential and microbial communities upon Si addition should also be considered (Khan et al., 2021). Apart from Si addition to soil, foliar Si application has good prospects considering the promising results in cucumber plants (Hernández-Apaolaza et al., 2020).

Coskun et al. (2019) raised an intriguing question of whether Si addition has any effect on the physiology of plants grown under optimal conditions. Regarding Fe status in optimally nourished plants, a unanimous conclusion cannot be drawn. Silicon supply produces diverse effects in different plant species or cultivars. Addition of Si to the Chinese long variety of cucumber grown in Fe sufficiency did not change any of the relevant parameters (Pavlovic et al., 2013). Somewhat different effects were noted after Si addition to the roots of another cucumber variety grown under optimal conditions. A slight decrease in chlorophyll content and shoot Fe concentration were observed in these plants (Hernández-Apaolaza et al., 2020). However, there were no significant differences in dry weight, ROS content, or lipid peroxidation level, suggesting that cell damage did not occur, and that plant growth was not hindered.

Several studies evidenced Si-induced Fe deficiency in optimally nourished rice. While the application of Si fertilizer improved the nutritional value of field-grown rice by increasing the concentrations of proteins and some mineral elements, it did not have the same effect on grain Fe concentration (Liu et al., 2017). This observation was supported by the assessment of the impact of Si on rice grown in hydroponics. According to Carrasco-Gil et al. (2018), Si supply decreased root Fe concentration and increased oxidative stress in rice grown under optimal Fe supply, resulting in enhanced translocation rate to the aerial part. Recently, Martín-Esquinas and Hernández-Apaolaza (2021) found that although both Fe translocation factor and shoot Fe concentration decreased in Si-supplied rice, an increase in chlorophyll concentration was observed, while dry weight and lipid peroxidation level were unaffected. In another study (Becker et al., 2020), Si addition decreased Fe concentration in rice root apoplast and shoot. While it did not affect the chlorophyll content, it led to an upregulation of the full cascade of Fe homeostasis-related genes in the roots of optimally nourished plants. Aside from this report, data on the influence of Si on the expression of genes related to Fe homeostasis in plants grown under optimal conditions are scarce. Transcriptomics and proteomics analyses of optimally nourished plants with or without the supply of Si have yielded opposing conclusions (Coskun et al., 2019). There are still unresolved questions: Does Si modulate gene expression in non-stressed plants, and to what extent? Is Si’s influence on Fe homeostasis dependent on Fe acquisition strategy, Fe uptake efficiency, or the plants’ ability to take up Si? It would be interesting to examine Si-dependent changes in gene expression during Fe sufficiency preceding Fe withdrawal, especially in plant species shown to benefit from Si supply in Fe-limiting conditions. Upregulation of genes related to Fe uptake and mobilization during a period of Fe sufficiency could be the hallmark of a Si priming effect that could prepare plants for subsequent Fe shortage and explain some aspects of the mechanisms underpinning Si-driven amelioration of this type of stress. Although Si-induced modulation of gene expression could merely be the indirect consequence of physicochemical changes in plant tissues caused by Si, knowledge related to these molecular processes could be valuable for recreating a more comprehensive model of the Si mode of action.

### 2.1 Influence of Si on Fe acquisition

Because of the discrepancies concerning the effect of Si on plant Fe status and tolerance to Fe deficiency stress, the proposed mechanistic models explaining Si action are still not well accorded. To develop a unifying model of Si’s activity, the results obtained by various research teams should be compared with regard to the differences in methodology, experimental designs, parameter selection, and data presentation. This would allow for general conclusions and the proposal of mechanistic models that could explain both the similarities and dissimilarities of the effects of Si in different plant species.

The root apoplast is considered to play a crucial role in nutrient acquisition. Since Fe contained in the root apoplast comprises 80% of total root Fe (Bienfait et al., 1985), the influence of Si on the deposition and mobilization of Fe from this compartment is recognized as an important aspect of Si-dependent modulation of Fe uptake and assimilation. To obtain a universal explanation of Si-promoted amelioration of a wide range of biotic and abiotic stresses in plants, Coskun et al. (2019) postulated the apoplastic obstruction hypothesis. It is founded on the property of Si to be deposited in the form of SiO_2_ in the apoplast surrounding exodermal and endodermal root cells, promoting the formation of the Casparian band (CB) by stimulating suberin and lignin biosynthesis (Fleck et al., 2011; Fleck et al., 2015). Deposits of Si in the root cell wall of graminaceous plant species colocalize with polymerized lignin (Soukup et al., 2017; Soukup et al., 2019). Consequently, in the presence of Si, the apoplastic bypass route for metal ions is impaired, leading to decreased root-to-shoot translocation and reduced accumulation of metals in the shoot. Based on this hypothesis, Becker et al. (2020) and Hernández-Apaolaza et al. (2020) explained the Si-induced reduction in leaf Fe concentration in rice growing under optimal and high concentrations of Fe and in Fe-deprived cucumber. This view is supported by the observation that Si promotes and accelerates the formation of exodermal CBs in the roots of rice, maize and some other plant species (Fleck et al., 2015). Although endodermal CBs were assumed to play a central role in the formation of an effective barrier that prevents the unselective flux of ions from the apoplast to the vasculature and towards the shoot, exodermal CBs were also proposed to be a diffusion barrier for Fe flow into the apoplastic space (Hinrichs et al., 2017).

Both local and systemic signals have been shown to control the expression of the genes involved in Fe acquisition in the root. The concentrations of Fe in the shoot and phloem are recognized as crucial long-distance signals that regulate Fe deficiency response in the root (Gayomba et al., 2015; Khan et al., 2018). Hence, the Si-induced decrease in Fe concentration in rice shoots could be the main driving force that upregulates the entire set of Fe homeostasis-related genes and leads to increased production of Fe-chelating substances (Becker et al., 2020).

In the report of Carrasco-Gil et al. (2018), the Si-induced Fe deficiency response led to an increase in root-to-shoot Fe translocation in rice. It should be noted that the experimental setup of this study differed from the one used by Becker et al. (2020). In the former study, rice was subjected to Fe deprivation after preculture under Fe sufficiency, with or without Si supply. Iron was applied in the form of Fe (III)-EDTA at pH 7.5. Since the stability of this chelate is low at pH>6.5, Fe plaque formation was observed on the root surface. Iron plaques, which are depositions of metal-containing iron oxides and hydroxides, appeared on the surfaces of aquatic and wetland plant roots. These formations can act as a barrier but also as a buffer by limiting or enhancing plant uptake of metals and metalloids depending on different environmental factors (Tripathi et al., 2014). As determined by SEM-EDX and LA-ICP-MS analyses, under Fe sufficiency, Si increased Fe deposition in plaques and decreased it in root hairs, root endodermis and the root vascular cylinder of rice (Carrasco-Gil et al., 2018). Thus, Fe concentration was decreased in plant tissues, which led to the activation of the Fe deficiency response and elevated root-to-shoot Fe translocation. The stimulation of these processes, in plants supplied with Si, continued when Fe was withdrawn from the nutrient solution, leading to enhanced Fe absorption from plaques; the concentration of Fe was lowered in plaques, whereas it increased in endodermal and vascular cylinder cells of roots. Total root and apoplastic Fe concentrations were lower in Si-treated plants. Changes in chlorophyll concentration and other parameters that determine the severity of Fe deficiency stress were not reported in this publication. Thus, it cannot be concluded whether the Si-provoked increase in Fe translocation to the shoot resulted in the alleviation of the stress, or whether it was not sufficient to produce such an effect. In a recent study, it was found that Si, in both acidic and calcareous conditions, lowers the Fe concentration in shoots of the treated rice (Martín-Esquinas and Hernández-Apaolaza, 2021). Nonetheless, Si exerted a beneficial effect, significantly increasing dry weight and chlorophyll concentration (i.e. SPAD index) of the Fe-deprived plants, especially at pH7.5. In the same paper, Si was linked to yet another important effect. Namely, the addition of Si enhanced endoreplication, i.e. the percentage of cells in the higher ploidy levels. This phenomenon has been suggested as an adaptive plant strategy to maintain the leaf size under environmental stresses.

Unlike the Si-induced decrease in apoplastic Fe concentration in rice grown under sufficient availability of Fe (as shown in Carrasco-Gil et al., 2018 and Becker et al., 2020), Pavlovic et al. (2013) reported increased apoplastic Fe concentration in cucumber roots supplemented with Si during pre-culture under Fe sufficiency. The withdrawal of Fe from the nutrient solution decreased the concentration of apoplastic Fe, and this process was accelerated in plants provided with Si. This was accompanied by a Si-induced increase in xylem Fe concentration in Fe-deprived plants, resulting in enhanced root-to-shoot Fe translocation and, consequently, in an increase in shoot Fe concentration and alleviation of dry biomass loss and leaf chlorosis. Silicon is considered to contribute to the crosslinking of cell wall structures that contain negatively charged sites capable of binding metal cations (Schwarz, 1973; Pavlovic et al., 2013). Also, the polymerization of orthosilicic acid in plant cell walls generates polysilicate that can bind metal ions as chelate-like complexes (Iler, 1979). Therefore, a possible mechanism of Si-induced increase in the pool of root apoplastic Fe in plants fed with a sufficient amount of Fe could provide additional Fe^3+^ binding sites in the root apoplast. As regards the subsequent Si-enhanced mobilization of Fe from the apoplastic pool in Fe-deficient conditions, a significant role was suggested for Fe-mobilizing compounds. Phenolic and flavin-like compounds secreted from root cells can chelate Fe from the root apoplast, rendering it available for translocation to the shoot (Rodríguez-Celma et al., 2011). Silicon-stimulated accumulation of flavonoid-type phenolics, gallic acid and riboflavin, as observed in the apical root tissue of Fe-deprived cucumber, is considered to have an important role in the reutilization of apoplastic Fe^3+^. Moreover, gallic acid and riboflavin could also enhance Fe^3+^ reduction to Fe^2+^, which is a required step in Strategy I Fe acquisition. Furthermore, Si stimulated Fe^3+^ chelate-reducing capacity and the expression of Strategy I Fe acquisition genes (Pavlovic et al., 2013).

### 2.2 Influence of Si on Fe remobilization

Root-to-shoot translocation as well as remobilization of Fe from old to young leaves is an important aspect of Fe-deficiency stress tolerance. Thus, special attention should be given to the role of these processes in the Si-driven alleviation of this type of nutritional stress. Knowledge on this subject could also guide further applicative research toward Si-aided mineral biofortification.

Fe-chelating compounds, including carboxylates and non-proteinogenic amino acids that increase Fe availability in plant tissues and mobility between plant organs, are of crucial importance for root-to-shoot translocation and Fe status in sink organs. Silicon-stimulated accumulation of Fe chelators and the upregulation of the expression of genes involved in their biosynthesis are recognized as an important feature of Si-induced amelioration of Fe-deficiency stress. This is supported by the finding that the addition of Si increased the concentrations of carboxylates, citrate and malate in cucumber root tissues, accompanied by an enhanced concentration of citrate in xylem sap (Pavlovic et al., 2013). While citrate is considered a major candidate for Fe xylem transport (Rellán-Álvarez et al., 2010), in the phloem and symplast compartment, Fe is predominantly complexed with NA, a plant-specific non-proteinogenic amino acid (Rellán-Álvarez et al., 2008). Since the xylem is underdeveloped in young leaves, their nutrient supply is dependent on phloem transport. Therefore, increased expression of the genes for NA biosynthesis and its accumulation in the shoots of Si-fed cucumber could be a prerequisite for Si-mediated Fe retranslocation from old leaves to young cucumber leaves, the organs most severely threatened by Fe deficiency (Pavlovic et al., 2016). Moreover, Si supply upregulates the expression of the gene encoding for the YSL1 transporter, which is responsible for phloem loading and unloading of the Fe-NA complex.

Aside from their Fe acquisition strategy, graminaceous plants differ from dicotyledonous plants in the way they accomplish Fe distribution. While in dicots old leaves are major source organs, in Gramineae, Fe retranslocation from the root to young leaves *via* the phloem is considered an important route for Fe remobilization (Tsukamoto et al., 2009). Silicon addition to Fe-deprived barley plants improved the Fe content in the youngest leaves owing to the root Fe content, while the content of the oldest leaves did not change substantially (Nikolic et al., 2019). Aside from direct Fe retranslocation from the root to young leaves, it is also possible that Fe is first translocated from the root to the first leaves at the beginning of the stress period when only the first leaves are fully expanded. Subsequent remobilization of the Fe from this pool also impacts the Fe status of younger developing leaves. Silicon-induced changes in the expression of Strategy II genes were in agreement with the observed effects. In barley root, Si supply accelerated the Fe deficiency-induced increase in the expression of the gene encoding for NA synthase (HvNAS1) and genes involved in phytosiderophore DMA (deoxymugineic acid) synthesis and uptake of Fe-DMA complexes (HvDMAS and HvYS1). In the leaves, Si caused a remarkable increase in *HvNAS1* and *HvTOM1* genes involved in the biosynthesis and transport of DMA, an Fe chelator specific to graminaceous plants, which pointed to its participation in Si-mediated amelioration of Fe deficiency.

**Figure 1:**
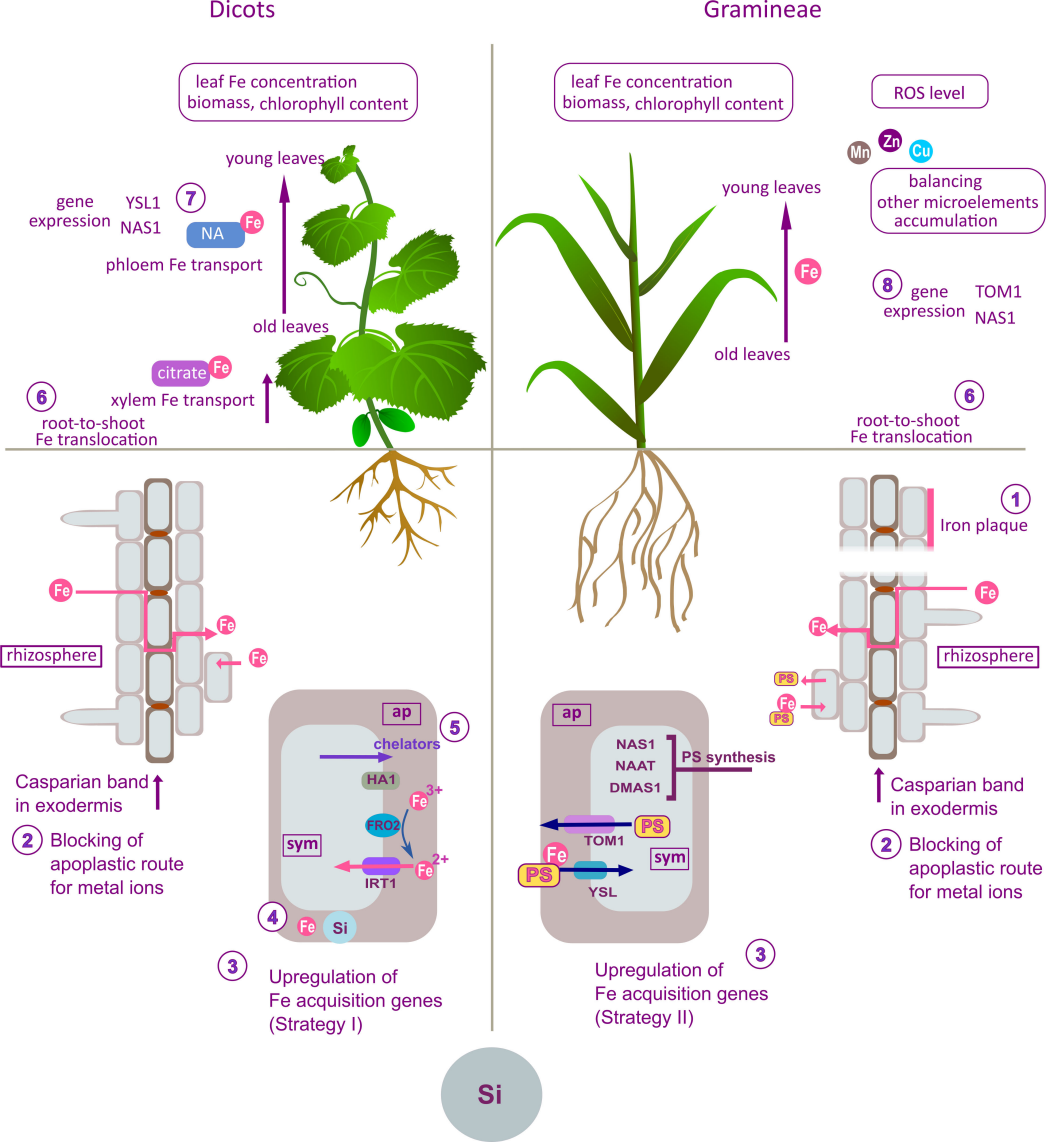
Summary of the effects of Si on Fe uptake, distribution and homeostasis in plants; proposed mechanisms obtained from differently designed experimental studies on different plant species or cultivars (left panel – dicots; right panel – Gramineae). The effects and proposed modes of action of Si are the outcome of compilation and review and are not combined in a unique model, nor given as canonical mechanisms of Si action. Si was found to increase the deposition of Fe on the root surface (1) in rice (Carrasco-Gil et al., 2018) or to promote formation of physical barriers in the root, impairing the apoplastic bypass route for Fe (2) in rice (Becker et al., 2020) and cucumber (Hernández-Apaolaza et al., 2020). As a result, the concentration of Fe decreased in plant tissues, leading to upregulation of Fe homeostasis-related genes (3) (Becker et al., 2020). On the other hand, according to Pavlovic et al., 2013, Si increased apoplastic Fe concentration (4) in cucumber roots during Fe pre-culture. After withdrawal of Fe from the nutrient solution, Si enhanced the mobilization of Fe from the root apoplastic pool by stimulating synthesis of Fe-chelating compounds (5) and upregulating Fe acquisition genes (3). Si-induced accumulation of Fe chelators (citrate) in xylem facilitated root-to-shoot Fe translocation (6), resulting in increased leaf Fe concentration and alleviation of a biomass loss and chlorosis. In shoot, Si increased Fe re-translocation from old to young cucumber leaves by stimulating biosynthesis of NA (7) Pavlovic et al., 2016. Si-promoted Fe translocation from root to young leaves (6) in some Gramineae species, resulted in increased Fe concentration and alleviated chlorosis, biomass loss and oxidative stress in young leaves (Nikolic et al., 2019; Teixeira et al., 2020). Silicon modulated expression of Strategy II Fe acquisition genes in root (3) and shoot (8) (Nikolic et al., 2019). Silicon reduced the accumulation of other microelements in youngest leaves by retaining them in the root (Nikolic et al., 2019). ap, apoplast; sym, symplast; PS, phytosiderophores.

The effects of Si on Fe uptake, distribution and homeostasis in plants, as well as the proposed underlying mechanisms reported in experimental studies with different designs on different plant species or cultivars, are summarized in **Table 1** and **Figure 1**.

**Table 1:** Effect of Si on plants under Fe deficiency and under optimal Fe supply, reported to date.

Effect of Si	Predominant strategy for Fe acquisition	Plant species and Si accumulation	Experimental design (in brief)	Effect of Si on Fe deficiency symptoms		Role of Si in Fe acquisition	Role of Si in Fe remobilization	Reference
				under Fe deficiency	in optimal Fe supply			
**beneficial**	Strategy I	*Cucumis sativus* L. cv Chinese long; moderate Si accumulator	Hydroponics; different experimental setups, -/+ Si/Fe preculture preceding Fe withdrawal; Fe supplied as Fe(III)-EDTA at pH6.0	diminished chlorosis and biomass loss	increased root apoplastic Fe concentration; chlorosis and biomass not affected	enhanced mobilization of Fe from the root apoplastic pool in Fe deprived plants; up-regulated expression of genes involved in Fe uptake and genes responsible for biosynthesis of Fe-mobilizing compounds	increased xylem Fe concentration resulting in an improved root-to-shoot Fe translocation; enhanced remobilization of Fe from older to younger leaves by a more efficient Nicotianamine (NA)-mediated Fe transport via the phloem	Pavlovic et al. (2013); Pavlovic et al. (2016)
		*Glycine max L.* cv. Klaxon	Hydroponics; CaCO3 added to simulate the calcareous soil conditions. pH adjusted initially to 7.0-7.5. Fe supplied as Fe(III)-HBED Different Si doses applied either at the beginning or continuously during the assay.	diminished chlorosis and biomass loss only when Si was supplied in an intermediate dose (0.5 mM)	when Fe was added as Fe(III)-EDTA, chlorophyll content and biomass were lowered in Si supplied plants; when Fe was added as Fe(III)-HBED leaf dry weight and Fe content were increased in the presence of Si.	N/A	N/A	Gonzalo et al. (2013)
		*Cucumis sativus* L., cv. Phoenix moderate Si accumulator	Hydroponics; 7 days pretreatment of Fe supply as Fe(III)-EDTA at pH 5.0 and 6.0 Si added after pretreatment.	diminished chlorosis and biomass loss especially in plants grown at pH 6.0	biomass not affected, increased Fe concentration in xylem sap and young leaves at pH5 of nutrient solution	increased Fe concentration and content in young leaves at pH6 of nutrient solution	N/A	Bityutskii et al. (2018)
	Strategy II	*Hordeum vulgare* Si accumulator	Hydroponics, 4 days pretreatment of Fe supply, Si addition after Fe removal	diminished chlorosis and biomass loss of the youngest leaves	N/A	accelerated increase in the expression of genes related to Strategy II	increased Fe concentration in the youngest leaves, lowered in root	Nikolic et al. (2019)
		*Sorghum bicolor* Si accumulator	Plants cultured in pots filled with washed sand; Si supplied via nutrient solution, during 2 weeks of pretreatment and 5 weeks of Fe def. treatment. Fe supplied as Fe-EDDHA	diminished chlorosis and biomass loss, decreased level of malondialdehyde (MDA)	no effect on Fe accumulation, MDA level and chlorophyll content	decreased Fe uptake efficiency under Fe deficiency	increased efficiency of translocation and utilization of Fe (compared to -Si plants under Fe deficiency)	Teixeira et al. (2020)
		*Oryza sativa L.* Si accumulator	Plants cultivated in hydroponics at pH 5.5, fixed with 1 mM MES or at pH 7.5, fixed with 0.3 mM HEPES Fe supplemented as Fe-EDTA. After 15 days of preculture, Fe was removed from the solution.	alleviated chlorosis and decreased MDA concentration, despite slightly decreased shoot Fe concentration; increased shoot dry weight and the percentage of cells in higher ploidy levels at pH 7.5	decreased shoot Fe concentration at both pH conditions	decreased Fe uptake	In Fe sufficiency, Si decreased Fe translocation factor at pH 7.5. In Fe deficiency, Si increased Fe translocation factor at both pH.	Martín-Esquinas and Hernández-Apaolaza (2021)
**without effect or enhancing stress**	Strategy I	*Cucumis sativus* cv. Ashley moderate Si accumulator	Hydroponics; CaCO3 added to simulate the calcareous soil conditions. Initially, pH adjusted to 7.0-7.5. Fe supplied as Fe(III)-HBED. Different Si doses applied either at the beginning or continuously during the assay.	delayed decrease of stem dry weight, stem length, and node number, but without chlorosis alleviation; reduced leaf Fe concentration, increased ROS and lipid peroxidation levels in young leaves; chlorophyll concentration and dry weight of young leaves not affected	N/A	N/A	N/A	Gonzalo et al. (2013)
		*Cucumis sativus* cv. Ashley moderate Si accumulator	Si addition to roots: Nutrient solution similar to the one in Gonzalo et al. (2013). 7 days of +Fe preculture, 7 days of Fe deficiency, followed by Fe resupply. Foliar Si application: 1.5 mM Si(OH)4 solution at pH 5.0 sprayed onto leaves	Si addition to roots: decreased Fe concentration in shoot, no significant differences in Fe concentration, dry weight and chlorosis of the young leaves; Foliar Si application: no significant differences in the dry weight and chlorosis. Plant recovery after Fe resupply was more effective in the Si-treated plants.	Si addition to roots: induced chlorosis, decreased Fe concentration in the old leaves; Foliar Si application: chlorophyll level and Fe concentration not affected	N/A	N/A	Hernández-Apaolaza et al. (2020)
	Strategy II	*Oryza sativa L.* Si accumulator	Fe deprivation after a preculture in Fe sufficiency, with or without Si supply. Fe applied in the form of Fe (III)-EDTA at pH 7.5 (fixed by by the addition of 3 mM HEPES)	decreased total root Fe concentration; changed micronutrient localization in roots; leaf Fe concentration, chlorophyll content and other parameters determining the severity of Fe deficiency stress not reported	decreased total root Fe concentration, increased oxidative stress; increased Fe deposition in plaques, while decreasing it in the root hairs, root endodermis, and root vascular cylinder	Under Fe sufficiency, Si supply increased Fe root plaque formation. This plaque acts as a barrier, reducing Fe uptake. Under Fe deficiency, +Si plants absorbed Fe from the plaque more efficiently than –Si plants	increased Fe translocation factor (ratio shoot/root concentration) under optimal Fe supply and in Fe deficiency	Carrasco-Gil et al. (2018)
		*Oryza sativa* Si accumulator	Hydroponics; 28 days treatment of low, optimal and high Fe supply, with or without Si addition. Fe supplied as Fe-EDDHA (or Fe (III)-EDTA or FeIISO_4_ for the Fe form experiments) at pH6.0	enhanced chlorosis; Fe concentration not significantly changed	in plants grown in excess Fe or under optimal Fe supply: chlorophyll content not affected; decreased Fe concentration in root apoplast and shoot	upregulation of the full cascade of Fe-homeostasis-related genes in the root	N/A	Becker et al. (2020)

N/A - not applicable.

## 3 Alleviatory effects of Si on plants exposed to toxic Cu

The role of Si in protecting plants from Cu toxicity has been studied less than its alleviatory effect on plants exposed to an excess of other essential and non-essential elements such as Zn, Cd and Al.

In the last few years, our understanding of the mechanisms of Si action in plants exposed to toxic Cu has increased. The first report on the potential beneficial effect of Si on Cu-stressed plants was reported by Nowakowski and Nowakowska (1997). In this study, Si stimulated biomass production in spring wheat treated with different concentrations of Cu. In addition, the increased water content in the roots and shoots was observed in seedlings grown with Si, and this effect was more prominent at higher concentrations of Cu (Nowakowski and Nowakowska, 1997). Silicon-stimulated protection of plants from elevated Cu stress has been shown for other plant species, including the Si-hyperaccumulator bamboo, the moderate Si accumulator cucumber, but also in two highly Cu-tolerant plant species, *Spartina densiflora* and *Erica andevalensis* (Oliva et al., 2011; Collin et al., 2014; Mateos-Naranjo et al., 2015; Bosnić et al., 2019b). Copper toxicity symptoms, root browning and chlorosis, were diminished in Si-fed bamboo, although plant biomass and the relative water content in leaves, stems or roots were not improved (Collin et al., 2014).

In cucumber, Si stimulated the growth of plants exposed to a range of doses of Cu (Bosnić et al., 2019b). The alleviatory effect of Si on leaf biomass loss was more pronounced than in the roots of Cu-treated cucumber. Accordingly, Si mitigated leaf chlorosis, which is one of the most prominent effects of Cu toxicity (Bosnić et al., 2019b). It was suggested that one important beneficial mechanism of action of Si is *via* elevation of the photosynthetic rate in Cu-stressed plants. Silicon amendment to the highly Cu-tolerant grass *Spartina densiflora* attenuated Cu-induced inhibition of RUBISCO enzyme activity and increased the concentration of photosynthetic pigments, resulting in a higher photosynthetic rate and plant biomass (Mateos-Naranjo et al., 2015).

Although no significant improvement in root biomass of Cu-treated plants was observed in the presence of Si, cell structure damage was significantly mitigated in rice roots, thus reducing uncontrolled Cu uptake (Kim et al., 2014).

In line with these effects of Si is the mitigation of oxidative stress damage, which manifests as a decrease in lipid peroxidation levels in cucumber and cotton exposed to elevated Cu concentrations (Ali et al., 2016; Bosnić et al., 2019b). Decreased oxidative stress in plants supplemented with Si are likely to be the consequence of reduced Cu uptake and lowered Cu accumulation in plant tissues. Additionally, it has been shown that Si stimulates antioxidative enzyme activities, and increased activities of SOD, catalase and ascorbate peroxidase have been observed in cucumber, cotton, flax and rice supplemented with Si during Cu-induced stress (Kim et al., 2014; Ali et al., 2016; Bosnić et al., 2019b; El-Beltagi et al., 2020; Riaz et al., 2022; Vieira-Filho and Monteiro, 2022). Although there is a number of publications demonstrating the role of silicon in mitigating oxidative stress caused by copper toxicity, little is known about the signaling underlying this effect. In the last year, two studies have been published that link nitric oxide (NO) and the beneficial effect of silicon in the stress of excess copper. First, the study of Si-mediated alleviation of Cu toxicity in mung bean seedlings clearly revealed that endogenous NO increases the viability of root cells by rendering them more tolerant to oxidative stress (Gaur et al., 2021). Second, in Salvia officinalis Cu stressed and control plants, both NO and Si were found to upregulate the genes and enzyme activity, demonstrating synergistic modulation of the secondary metabolism. (Pirooz et al., 2022). In this study, PAL enzyme activity was elevated upon Si and NO addition, leading to an increased synthesis of polyphenols that have the potential to scavenge harmful reactive oxygen species). These initial studies are clearly promising, but additional studies are needed to fully characterize the role of endogenous NO and other signal molecules in Si-governed mitigation of Cu toxicity.

### 3.1 Influence of Si on Cu uptake

The addition of Si also influences Cu accumulation in plants. In seedlings of spring wheat, Si strongly reduced the Cu content (Nowakowski and Nowakowska, 1997). A similar effect was observed in other plant species, including cucumber, rice, cotton and zinnia (Frantz et al., 2011; Kim et al., 2014; Ali et al., 2016; Bosnić et al., 2019b). In contrast, such an effect was not detected in bamboo, a Si hyperaccumulator (Collin et al., 2014), nor in Arabidopsis, a species classified as a Si excluder (Li et al., 2008), indicating species specificity of the phenomenon rather than its dependence on the amount of Si present in the tissues.

Silicon-dependent regulation of genes involved in Cu homeostasis and restriction of Cu uptake correlates with a decrease in Cu concentration in the roots of cucumber and tobacco (Flora et al., 2019; Bosnić et al., 2019b, respectively). One of the most important regulators of Cu uptake in roots is the plasma-membrane high affinity Cu^+^ transporter COPT1 (Sancenón et al., 2004). Copper treatment downregulated *COPT1* in tobacco, and provision of Si additionally decreased the expression of this gene (Flora et al., 2019).

Investigation of the influence of Si on the transport function of COPT1 and other participants in Cu uptake (e.g. FRO4 reductase), including a transgenic approach, should be considered in order to demonstrate the proposed role of this Cu transport route in the ameliorative effect of Si on Cu-treated plants.

### 3.2 Influence of Si on Cu distribution in plants

Silicon influences root-to-shoot translocation and alters Cu deposition in leaves. A lower Cu concentration in the leaves of *Erica andevalensis*, a species tolerant to high Cu content, was observed in the presence of Si. This effect was explained by the inhibition of Cu translocation from the roots to the leaves and its retention in roots (Oliva et al., 2011). Prevention of metal translocation might be the result of Si-stimulated formation of the Casparian band as well as enhanced lignin deposition in root endodermis, which serves as a physical barrier to an unselective heavy metal flux (Vaculík et al., 2012). The effect of Si on decreased Cu translocation in durum wheat was attained *via* Si-stimulated endodermis thickening and increased Cu adsorption on the root surface, and Cu immobilization in the epidermis (Keller et al., 2015). In contrast, Si did not influence the concentration of Cu in *Arabidopsis* leaves (Li et al., 2008).

Heavy metal stress alters the lignification process in plants. Lignin accumulation is accompanied by an increased production of precursors, phenolic compounds that are synthesized in the phenylpropanoid pathway where phenylalanine ammonia-lyase (PAL) is one of the key enzymes (Dixon and Paiva, 1995; Frei, 2013). The intensity of Cu stress has been shown to positively correlate with the content of phenolics, lignin accumulation and PAL expression or activity (Ali et al., 2006; Kováčik and Klejdus, 2008; Pirooz et al., 2022).

In cucumber roots, Cu treatment causes intensive lignification, while provision of Si decreased lignification and lowered the level of *CsPAL1* expression and the total content of phenolics (Bosnić et al., 2019b). Lowered lignification in the roots of cucumber in the presence of Si indicates alleviated stress, which is confirmed by decreased lipid peroxidation in cucumber roots (Bosnić et al., 2019b). Similarly, reduced expression of four PAL genes was detected in *Arabidopsis* shoots (Li et al., 2008; Khandekar and Leisner, 2011). According to these findings, lignification is not recognized as a mechanism by which Si decreases Cu toxicity in cucumber and Arabidopsis, and other mechanisms should be considered to explain the beneficial effect of Si.

Most of the Cu in the plant cell is bound to cell wall components, with up to 60% of the total Cu content being immobilized and therefore less toxic to the cell protoplast (Iwasaki et al., 1990; Krzesłowska, 2011). In cucumber roots, Si increased the deposition of Cu in the cell walls and lowered the total Cu content inside the plant cells (Bosnić et al., 2019b). Silicon-induced deposition of metals in the cell wall is also considered an important route of protection from other potentially toxic metals such as Al, Cd and Mn (Wang et al., 2004; Dragišić Maksimović et al., 2012; Vaculík et al., 2012).

In the roots, Si-stimulated Cu deposition in the cell wall is important for both the protection of root cells and retention of Cu excess in the root apoplast, aimed at reducing root-to-shoot Cu translocation. The resulting decrease in Cu accumulation in the leaves is accompanied by another protective mechanism that occurs in the leaf tissue itself. Silicon and copper co-deposition in the leaf phytoliths was proposed to participate in the alleviation of the symptoms of Cu toxicity in *Erica andevalensis* (Oliva et al., 2011). However, Cu and Si co-localization in root cell walls and leaf phytoliths was not observed in other species such as durum wheat (Keller et al., 2015), indicating that these mechanisms of Si action should not be considered as widespread.

### 3.3 Effects of Si on Cu-binding proteins

Half of all the Cu in green tissue cells is bound to three proteins: CSD1, CSD2 and plastocyanin (PC) (Abdel-Ghany, 2009). Since a relatively small amount of functional plastocyanin is sufficient for maximum photosynthesis intensity, additional accumulation of plastocyanin actually occurs in response to the presence of excess Cu (Abdel-Ghany and Pilon, 2008; Abdel-Ghany, 2009). In addition, plastocyanin can bind free Cu ions *in vitro* and thereby reduce the production of a harmful hydroxyl radical (Zhou et al., 2018). Bosnić et al. (2019b) showed that the amount of plastocyanin in plants treated with Cu increases to a much greater extent if the plants are grown in the presence of Si. This study showed for the first time that the application of Si stimulates the accumulation of PC that binds and stores excess Cu in the leaves and consequently reduces the risk of the effects of reactive Cu ions, contributing to better plant performance (Bosnić et al., 2019b).

Cytosolic CSD1 and plastidic CSD2 are considered as the most important isoforms of SOD, with a central role in plants’ response to stress (Alscher et al., 2002; Abdel-Ghany and Pilon, 2008). In cucumber plants, Si supply raised the root and leaf protein levels of both CSD isoforms, which was in accordance with its effect on overall SOD activity. Therefore, it can be concluded that stimulation of SOD expression is an important component of the Si-mediated mechanism of plant tolerance and stress reduction (Bosnić et al., 2019b).

### 3.4 Effects of Si on Cu ligands and chelators

Different organic molecules are involved in the maintenance of metal homeostasis, including thiol compounds (S-ligands), organic acids (O-ligands) and amino acids (N-ligands) (Hall, 2002; Sharma and Dietz, 2006; Haydon and Cobbett, 2007; Anjum et al., 2015).

Metallothioneins and phytochelatins are important S-ligands in plants with a high affinity for binding Cu (Mijovilovich et al., 2009; Carrillo and Borthakur, 2021). The expression of *MT2a* and *MT2b* in *Arabidopsis* leaf was stimulated by Si (Khandekar and Leisner, 2011). Spectroscopic methods have confirmed that Si promotes Cu binding to S-ligands in bamboo leaves (Collin et al., 2014). Authors suggested that these changes enabled plants to respond to Cu toxicity more effectively.

Silicon also affects the accumulation of organic acids such as citrate, malate and aconitate. Their role is important not only for the transport of metals, but also for the tolerance to metals that are present in excess (Küpper et al., 2009). In addition to the accumulation of citrate and malate, Si influenced aconitate accumulation in cucumber plant leaf (Bosnić et al., 2019b). The formation of Cu complexes with organic acids, especially with aconitate, is among the most important mechanisms in the beneficial effects of Si in wheat (Keller et al., 2015).

Amino acids such as His and NA form very stable complexes with Cu, and their synthesis is induced in the presence of high concentrations of Cu in plants (Liao et al., 2000; Irtelli et al., 2008). Nicotianamine, being a high-affinity ligand, has a unique role in Cu homeostasis (Pich and Scholz, 1996; Takahashi et al., 2003), and the accumulation of NA is the main strategy for detoxification in plants that are not tolerant to the presence of excess metals (Mijovilovich et al., 2009; Anjum et al., 2015). Conversely, His is a chelating amino acid responsible for metal hypertolerance and hyperaccumulation in many plant species (Krämer and Clemens, 2006). Copper complexes with amino groups were detected in Cu-treated bamboo, as well as an increased accumulation of His in the roots of plants grown in the presence of Si (Collin et al., 2014). Increased concentrations of NA and His were measured in cucumber leaves after treatment with excess Cu, and the application of Si additionally enhanced their accumulation (Bosnić et al., 2019a). Chelation of Cu ions by NA and His is a mechanism by which the concentration of free ions are effectively controlled, resulting in plant tolerance to the metals present in excess (Bosnić et al., 2019a).

**Figure 2:**
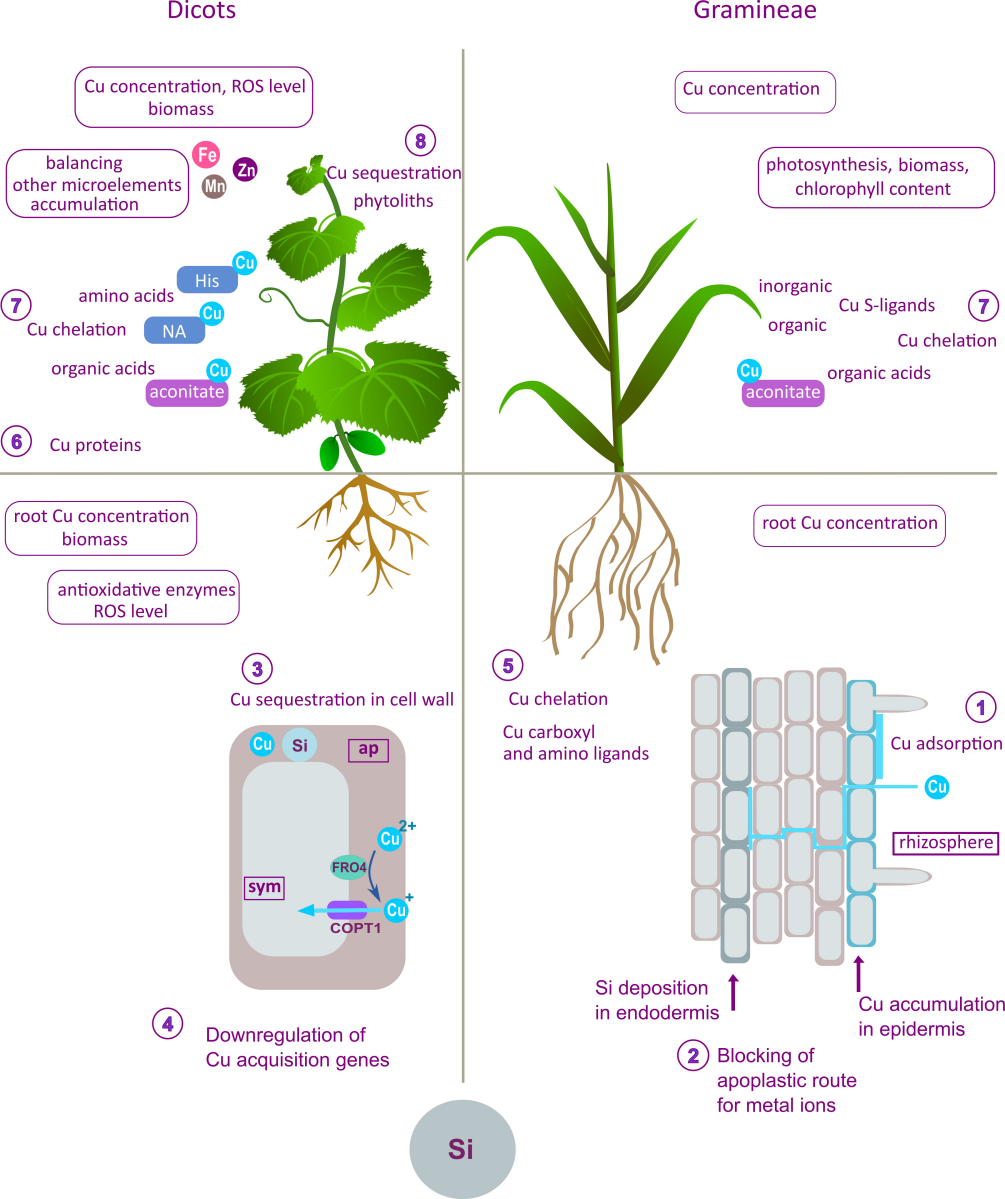
Summary of the effects of Si on Cu uptake, distribution, and homeostasis proposed mechanisms obtained from differently designed experimental studies on different plant species or cultivars (left panel – dicots; right panel – Gramineae). The effects and the proposed modes of action of Si shown in this figure are the outcome of compilation and review and are not combined in a unique model, nor given as canonical mechanisms of Si action. The beneficial effects of Si at the root level include preservation of root structures (Kim et al., 2014) as the consequence of several proposed mechanisms. In durum wheat, Si increased adsorption of Cu onto the root surface (1), enhanced endodermis thickening and Cu retention in the epidermis, thereby limiting Cu translocation (2) (Keller et al., 2015). In dicots, decreased Cu accumulation and lowered oxidative stress in Si-treated plants are the consequence of Cu-sequestration in root cell walls (3) and downregulation of the genes involved in Cu uptake (4) (Flora et al., 2019; Bosnić et al., 2019b). Si-promoted Cu fixation in roots prevents root-to-shoot translocation and lowers Cu content in leaves (Oliva et al., 2011). Copper complexation by amino and carboxyl ligands (5) in bamboo root decreased visible toxicity symptoms in plants grown with Si (Collin et al., 2014). Alleviative mechanisms in the leaf include enhanced Cu complexation by Cu-proteins (Cu/Zn SOD, plastocyanin) (6); organic acids (aconitate) and amino acids (NA, His) (7) which were detected in cucumber leaves (Bosnić et al., 2019a; Bosnić et al., 2019b). Aconitate is also an important part of Si protection in durum wheat (Keller et al., 2015). Silicon stimulated Cu-binding to organic and inorganic S-ligands in bamboo leaves (7) (Collin et al., 2014). Copper sequestration into phytoliths (8) contributes to Cu tolerance in Cu-tolerant species (Oliva et al., 2011). Si-stimulated antioxidative activity was found to mitigate oxidative stress in several species (Khandekar and Leisner, 2011; Ali et al., 2016; Bosnić et al., 2019b). Maintenance of a higher photosynthetic rate enabled higher biomass production and reduction in leaf chlorotic symptoms (Mateos-Naranjo et al., 2015). A significant effect of Si was lower Cu concentration in the leaf (Nowakowski and Nowakowska, 1997; Frantz et al., 2011; Oliva et al., 2011; Collin et al., 2014; Ali et al., 2016; Bosnić et al., 2019b). Additionally, Si reduced the antagonistic effect of excess Cu on Fe, Zn and Mn concentrations in leaf (Oliva et al., 2011; Mateos-Naranjo et al., 2015; Bosnić et al., 2019a).

The ameliorative effects and the proposed modes of action of Si in Cu toxicity in plants are presented in **Table 2** and **Figure 2**.

**Table 2:** Si effect on plants under Cu toxicity and in optimal Cu supply, reported to date (↑ stimulation, ↓ reduction, − no effect).

Plant species and Si accumulation	Cu toxicity symptoms			Cu aquisition			Metal concentration in leaf				Expression/ activity of PAL	Antioxodant enzyme activity	Expression of SOD	Cu chelators				NO production	Reference
	Root and leaf biomass	Chlorophyll content	Lipid peroxidation Malondialdehyde (MDA) concentration	COPT1 expression	FRO4 expression	Cu root concentration	Cu leaf concentration	Fe leaf concentration	Mn leaf concentration	Zn leaf concentration				Metallothionein expression	Phytochelatin synthase expression	Organic acid concentration	Nicocianamine concentration		
*Arabidopsis thaliana*	↑			↓		−	−	−	−	−	↓	↑	↑	↑	−				(Li et al., 2008; Khandekar and Leisner, 2011)
*Antirrhinum majus*	−					↓	−	−	−	−	−								(Frantz et al., 2011)
*Zinnia elegans*	↑					↓	↓	−	↑	−	↓								(Frantz et al., 2011)
*Erica andevalensis*	↑					↑	↓	−		↑									(Oliva et al., 2011)
*Phyllostachys fastuosa*	−					−	−												(Collin et al., 2014)
*Oryza sativa*	↑	↑	↓			↓													Kim et al., 2014
*Spartina densiflora*	↑/−	↑				−	↓		↑										(Mateos-Naranjo et al., 2015)
*Triticum turgidum*	↑	−				↓	↓		↓	↓						↑			(Keller et al., 2015)
*Gossypium hirsutum*	↑	↑	↓			↓	↓					↑							(Ali et al., 2006)
*Nicotiana tabacum*	↑			↓		↓	−							↓	↓				(Flora et al., 2019)
*Cucumis sativus*	↓	↑	↓	↓	↓	↓	↓	↑	↑	↑	↓	↑	↑	↓	↑	↑	↑		(Bosnić et al., 2019a; Bosnić et al., 2019b)
*Vigna radiata*	↑	↑	↓									↓						↑	(Gaur et al., 2021)
*Panicum maximum* cv. Tanzania			↓									↑							(Vieira-Filho and Monteiro, 2022)
*Salvia officinalis*	−/↑	↑									↑							↑	(Pirooz et al., 2022)

## 4 Influence of Si on the crosstalk of essential microelements

Redundancy and crosstalk between the metabolic pathways involving essential elements are especially evident for Cu and Fe, which may be explained by the similarity of their chemical properties (Schmidt et al., 1997; Michaud et al., 2008). For example, the Cu and Fe status and the Cu : Fe ratio are reflected on the SOD isoform that is preferably accumulated in plant tissue. Copper excess leads to Fe deficiency, while in Fe-deprived plants Cu concentration is increased (Schmidt et al., 1997; Michaud et al., 2008; Cai et al., 2021). Silicon improves the homeostasis of both elements, in Cu excess and in Fe deficiency stress.

Fe deficiency leads to the accumulation of other heavy metals (Baxter et al., 2008). An elevated uptake of other metals such as Cu is required to provide cofactors for isofunctional enzymes, to replace Fe-requiring forms of enzyme. However, under certain states of prolonged Fe deficiency, it results in the undesirable accumulation of heavy metals. It was suggested that downregulation of the Fe acquisition machinery occurs during prolonged Fe deficiency to prevent a disbalance of other micronutrients (Le et al., 2016). In line with this notion is the observation that one of the effects of Si in barley deprived of Fe was a diminished accumulation of Mn and Zn in the youngest leaves, while their content in the root was increased (Nikolic et al., 2019). Accordingly, in barley supplied with Si, the expression of Strategy II genes *HvNAS1*, *HvTOM1, HvDMAS* and *HvYS1*, in the roots was decreased below the control level during prolonged Fe-deficiency stress.

Copper in excess inhibits the uptake of other essential elements, Zn, Mn and Fe, because of competition for non-selective ion membrane transporters (Wintz et al., 2003). By altering Cu availability in the root apoplast and root-to-shoot translocation, Si influences the acquisition and homeostasis of other elements that are impaired in Cu stress. Silicon significantly improved Mn concentration, which was decreased in Cu-treated *Spartina densiflora* (Mateos-Naranjo et al., 2015). Copper stress-induced reduction of the Zn content in *Erica andevalensis* leaves was alleviated in the presence of Si (Oliva et al., 2011). Similarly, Si diminished the antagonistic effect of Cu on leaf accumulation of Fe, Zn and Mn in cucumber exposed to Cu excess (Bosnić et al., 2019a). Excessive amounts of heavy metals not only lead to decreased total concentrations of other micronutrients, but also to a disturbed molar ratio between the elements. The molar ratio of Fe to other elements has been found to be critical in triggering an Fe deficiency response (Kobayashi et al., 2012). In Cu-treated cucumber, the molar ratios of Fe : Cu, Zn : Cu and Mn : Cu were severely disturbed (Bosnić et al., 2019a). The improvement of Fe : Cu and Zn : Cu molar ratios by the supply of Si is consistent with the alleviation of stress symptoms observed in these plants

The antagonistic interaction between Cu and Fe can also be used to alleviate Cu toxicity symptoms (Ouzounidou et al., 1998). In the above-mentioned study (Bosnić et al., 2019a), it was shown that additional Fe supply reduced Cu accumulation and improved the micronutrient molar ratio in cucumber, and this effect was even more pronounced than that resulting from the addition of Si. However, the most effective treatment for restoring Fe, Zn and Mn content and Fe : Cu and Mn : Cu molar ratios was the simultaneous supply of additional Fe and Si.

Some of the Si modes of action are shared among different types of stress. It is suggested that the Si-mediated alleviation of chlorosis in Cu-treated cucumber is a consequence of the increased Fe concentration in the leaf (Bosnić et al., 2019a). Furthermore, Si induced synthesis of Fe chelators such as citrate and NA was detected in cucumber growing in low Fe nutrient medium, but also in Cu stress induced Fe deficiency (Pavlovic et al., 2013; Pavlovic et al., 2016; Bosnić et al., 2019a).

The expression of genes involved in nutrient uptake is influenced by both local and systemic signals. In addition, nutrient signalling is also thought to be interdependent. Chaiwong et al. (2020) demonstrated that the expression of the Si uptake transporter LSi1 is regulated not only by Si concentration but also by the availability of Fe. Iron deficiency led to upregulation of LSi1, showing complex coordination among the nutrient-derived signals.

## 5 Conclusions and further prospects

Significant advances in understanding the mechanisms of the effect of Si on plant nutrition and its modulation of plant responses to nutritional stresses have been made. However, some important issues have yet to be addressed and resolved.

There is a substantial body of evidence indicating that the expression of genes relevant to the uptake and translocation of Fe, Cu and other essential elements changes when Si is supplied. However, our mechanistic understanding of this influence is still scant. Evidence indicates that this effect is an indirect consequence of the physicochemical changes in plant tissues caused by Si, and it has been suggested that Si does not have a direct influence on the members of the signaling cascades involved in plant stress response (Coskun et al., 2019).

An important aspect of the ameliorative effect of Si is its influence on the cellular localization of microelements. Silicon promoted Cu deposition in root cell walls (Bosnić et al., 2019b), and Si-stimulated formation of the Casparian band and endodermis thickening as physical barriers for Cu flow (Keller et al., 2015) are important in reducing excessive Cu uptake. In contrast, the Si-induced increase in the root apoplastic Fe pool in plants fed with sufficient Fe and subsequent Si-enhanced mobilization of Fe from the apoplastic pool in Fe-deficient conditions have been suggested to play a major role in Si-mediated amelioration of Fe deficiency in some plants (Pavlovic et al., 2013). Conversely, the Si-promoted formation of the Casparian band is considered to impair the apoplastic bypass route for Fe, leading to decreased root-to-shoot translocation and upregulation of Fe deficiency response (Becker et al., 2020; Hernández-Apaolaza et al., 2020). Future investigations and standardization of experimental methods, experimental designs and choice of evaluated parameters should facilitate comparison of results obtained by different research teams on diverse plant species, and contribute to a more comprehensive understanding of the mechanisms of the mode of action of Si.

The crucial role of metal-chelating compounds in Si-mediated amelioration of microelement disbalance is recognized (Pavlovic et al., 2013; Bosnić et al., 2019a). Their contribution to the beneficial effect of Si is similar in Fe deficiency and Cu stress. Chelators are found to both improve the mobilization of a deficient element and scavenge excess heavy metals. Furthermore, the complexation of metals with Si itself is considered to be an important process for increasing the bioavailability of a deficient metal (Stevic et al., 2016).

The metabolic pathways involving Fe and Cu are linked as they compete for common metal transporters and metal-binding proteins. Excess Cu leads to a decrease in the overall concentration of other micronutrients, while Fe deficiency can cause increased accumulation of other heavy metals. In both Cu excess and Fe deficiency, Si improves the homeostasis of both elements as well as their molar ratio.

The ability of Si to stimulate Fe remobilization from pools and source organs to young developing plant parts *via* the phloem is of vital importance, not only for supporting plant growth under conditions of Fe deficiency, but also for increasing crop nutritional value. Applicative research that explores the potential of Si for grain biofortification should provide valuable information in the design and production of eco-friendly and cost-effective solutions for improving the nutritional content and quality of crops.

## Author contributions

All authors designed and structured the review, collected the information, organized the figures and tables, and wrote and revised the manuscript. All authors contributed to the article and approved the submitted version.
